# *Epilobium angustifolium* L. as a Potential Herbal Component of Topical Products for Skin Care and Treatment—A Review

**DOI:** 10.3390/molecules27113536

**Published:** 2022-05-31

**Authors:** Anna Nowak, Joanna Zielonka-Brzezicka, Magdalena Perużyńska, Adam Klimowicz

**Affiliations:** 1 Department of Cosmetic and Pharmaceutical Chemistry, Pomeranian Medical University in Szczecin, Powstańców Wielkopolskich Ave. 72, 70-111 Szczecin, Poland; joanna.zielonka-brzezicka@pum.edu.pl (J.Z.-B.); adklim@pum.edu.pl (A.K.); 2 Department of Experimental and Clinical Pharmacology, Pomeranian Medical University in Szczecin, Powstańców Wielkopolskich Ave. 72, 70-111 Szczecin, Poland; magdalena.peruzynska@pum.edu.pl

**Keywords:** *Epilobium angustifolium* L., skin, cosmetics, antioxidant, anti-inflammatory, anti-aging

## Abstract

*Epilobium angustifolium* L. (EA) has been used as a topical agent since ancient times. There has been an increasing interest in applying EA as a raw material used topically in recent years. However, in the literature, there are not many reports on the comprehensive application of this plant to skin care and treatment. EA contains many valuable secondary metabolites, which determine antioxidant, anti-inflammatory, anti-aging, and antiproliferative activity effects. One of the most important active compounds found in EA is oenothein B (OeB), which increases the level of ROS and protects cells from oxidative damage. OeB also influences wound healing and reduces inflammation by strongly inhibiting hyaluronidase enzymes and inhibiting COX-1 and COX-2 cyclooxygenases. Other compounds that play a key role in the context of application to the skin are flavonoids, which inhibit collagenase and hyaluronidase enzymes, showing anti-aging and anti-inflammatory properties. While terpenes in EA play an important role in fighting bacterial skin infections, causing, among other things cell membrane, permeability increase as well as the modification of the lipid profiles and the alteration of the adhesion of the pathogen to the animal cells. The available scientific information on the biological potential of natural compounds can be the basis for the wider use of EA in skin care and treatment. The aim of the article is to review the existing literature on the dermocosmetic use of *E. angustifolium*.

## 1. Introduction

**Figure 1:**
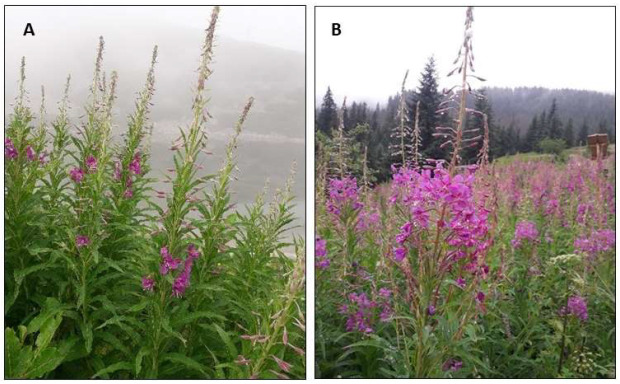
*Epilobium angustifolium* in a natural location in the Carpathians. (**A**)—Slovak Tatras, 1751 m a.s.l.; (**B**)—Polish Tatras, 1333 m a.s.l.

Fireweed (*Epilobium angustifolium* (L.) Holub) (Onagraceaeis) belongs to the genus *Epilobium*, consisting of more than 200 species [1,2,3] and occurs mainly in North America, Asia, and Europe [4,5]. Fireweed is also known as perennial, and the most commonly used names in the literature are the following: *Epilobium angustifolium* L., *Chamerion angustifolium* (L.) Holub and *Chamaenerion angustifolium* (L.) Scop., while it is colloquially used as perennial fireweed, narrow-leaved fireweed, great willowherb, willowherb, flowering willow, and french willow [6]. *Epilobium angustifolium* (EA) grows both in the lowlands and in the mountains [6] (Figure 1). In Northern and Eastern Europe, it is applied as a food plant, especially in the form of tea [5] or as a traditional herbal drug [7]. The great popularity of this plant is mainly due to its anti-inflammatory, antioxidant [8,9], antibacterial [10,11,12], and anti-cancer properties [13,14].

The historical sources say that this plant was once often used in various skin diseases [15]. However, there are not many reports in the available literature on a comprehensive description of this plant’s application for skin care, and its use in ready-made cosmetic or dermatological preparations is relatively small. In recent years, there has been an increased interest in dermatological preparations containing plant-derived ingredients. The preparations containing plant extracts, due to the high content of active ingredients, can exhibit multiple biological effects [8,16]. The aim of the article is to review the existing literature on the dermocosmetic use of *E. angustifolium*.

## 2. Chemical Composition

**Figure 2:**
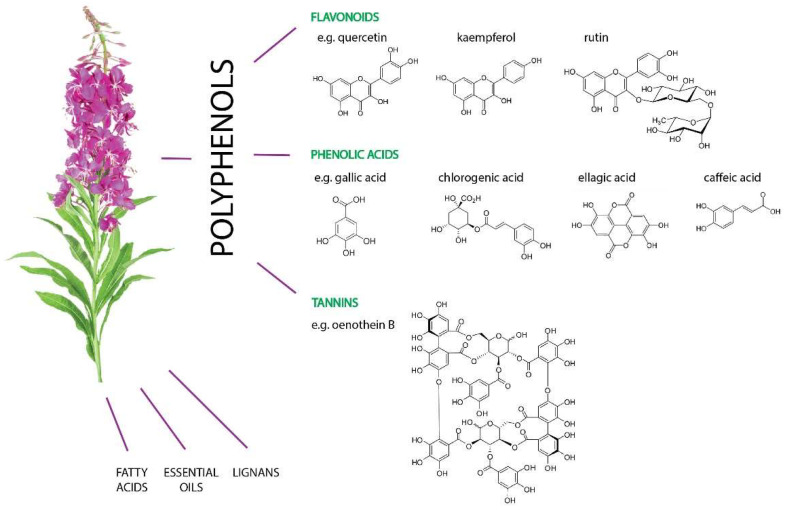
Groups of active compounds contained in *E. angustifolium* with examples of chemical structures. The main group is polyphenols including flavonoids, phenolic acids, and tannins. *E. angustifolium* is also a valuable source of fatty acids, essential oils as well as lignans.

Approximately 250 different secondary metabolites with possible biological activity have been identified in the EA [6] (Figure 2). The main components of EA are polyphenols, especially hydrolyzable tannins (ellagitannins), flavonoids, and phenolic acids [6,17,18,19]. The main phenolic acids are, among others, the following: gallic acid, 4-hydroxybenzoic acid, caffeic acid, chlorogenic acid, rosmarinic acid, ellagic acid, protocatechinic acid, and cinnamic acid [8,12,20,21], while the major flavonoids are the following: quercetin, myricetin, myricitrin, isomyricitrin, kaempferol, rutin, epicatechin hyperoside and isoquercetin [11,21]. Another important group is tannins, among others, oenothein B (OeB), which is mentioned as one of the main substances with the biological activity of this plant [6,20,22]. EA also contains a small amount of the essential oil in leaves and stems, composed mainly of terpenes such as anethole, β-bisabolene, α-caryophyllene, and caryophyllene, eugenol, linalool, pelargol, and menthol [6,23,24]. Several other groups of compounds such as lignans have also been identified, including pinoresinol, pinoresinol-4-O-glucopyranoside, 4-ketopinoresinol, as well as fatty acids, as follows: arachic acid, arachidic acid, myristic acid, oleic acid, palmitic acid, palmitoleic acid, stearic acid, octacosanic acid, nonacosanic, and 2-hydroxytriacontanic acid. Other compounds that can be characterized by high activity include L-ascorbic acid, charenol, chanerozan, choline, loliolide, and 2-hydroxybenzothiazole [6].

## 3. Ethnopharmacological Importance of *E. angustifolium* in Improving Skin Conditions

The *Epilobium* is from ‘Epi’ meaning upon, and Lobium pod, lobon, or capsule. Whereas the name *Chamerion* comes from the Greek ‘Chamae’ or ‘Khamai’ and means “on the ground,” and *Erion* means wool. However, according to other sources, the doctor and naturalist Gesner in the year 1561 named this plant fireweed (from the German: Feuerkraut), which means the plant that flourishes on ground cleared by fire. The Swedish doctor and naturalist Carl Linnaeus, during a famine in 1756, included the EA in the edible plants; whereas he reports on the 12 dialectical names, among others, the following: Weasel Milk, Elk Food, Heaven Grass [15]. The use of EA in the treatment of dermatological diseases has a very long tradition and is based mainly on historical or anecdotal evidence. The EA has been used to improve skin conditions since ancient times. The Canadian Indians (the Cree) macerated EA root and then applied it to boils and infections; moreover, the leaves as patches for bruises were used. The root has also been applied as an antiseptic to draw out infection from open wounds. Whereas the Algonquins used a freshly grated root as a poultice for boils [15]. The aerial parts of the EA have been used for a long time in various skin infections, mainly as an anti-inflammatory and antiseptic drug, as well as in the treatment of mycoses, burns, skin rashes, ulcerations, and wounds. An ointment made from the leaves was applied to treat skin diseases in children [25].

## 4. *Epilobium angustifolium* in Cosmetic and Dermatological Preparations

**Figure 3:**
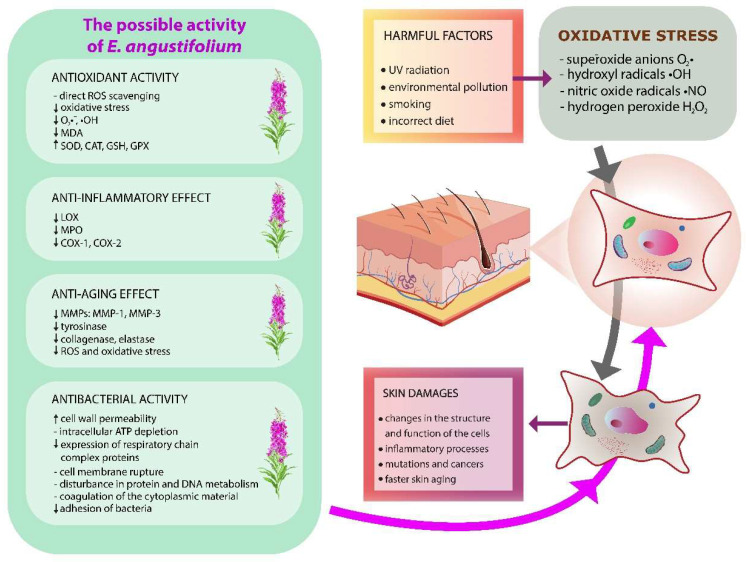
The possible effect of *E. angustifolium* on skin damage due to oxidative stress occurring. Bioactive compounds contained in *E. angustifolium* have an antioxidative, anti-inflammatory, anti-aging, as well as antibacterial potential. UV radiation, environmental pollution, smoking, or an incorrect diet may enhance the phenomenon of oxidative stress, which may result in changes in the cells’ structure and function, increase inflammatory processes, mutations, and cancerogenesis, or result in faster skin aging.

The skin is the largest living organ that protects the body against the external environment. The skin helps to regulate the temperature, performs a secretory function, protects against external toxic chemicals and harmful microorganisms, as well as protects against the sun’s rays [26]. The area of the skin of adults was 1.5–2.0 m^2^, on average, and its mass is approximately one-tenth of the human body weight. It consists of layers separated by a basal membrane, under which there is adipose and connective tissue (subcutaneous tissue). The epidermis consists of several types of cells, such as keratinocytes, melanocytes, Merkel cells, and Langerhans cells [27,28]. In recent years, an increasing interest in applying natural methods to the treatment and care of the skin has been observed. Patients often choose preparations containing ‘natural’ ingredients, which are perceived to be safer compared to ‘synthetic’ ingredients [9]. Due to the richness of plants in valuable secondary metabolites, the preparations containing plant extracts may be characterized by biological effects, i.e., antioxidant, anti-inflammatory, anti-aging, or antibacterial (Figure 3, Table 1).

### 4.1. Antioxidant Activity

The skin is constantly subjected to oxidative control caused by an imbalance between oxidants and antioxidants in favor of oxidants. This leads to a disruption of redox signaling and, as a consequence, molecular damage [31,32,33]. Moreover, the skin is attacked constantly by reactive oxygen species (ROS), which are continuously produced as secondary products of some metabolic pathways as well as by specific systems, such as the pathological inflammatory processes, oxidases, cytokines, peroxisomes, xanthine oxidase (XO), NADPH oxidase, acetyl CoA oxidase, or cytochromes [34]. The main ROS causing oxidative stress are superoxide anions (O_2_∙^−^), hydroxyl, nitric oxide radicals, and hydrogen peroxide (H_2_O_2_), which play a key role in oxidative stress. Excessive production of ROS can damage cells and tissues by modifying and damaging proteins, phospholipids, or nucleic acids. As a result, the structure of the cell membrane changes and its function, which disrupts vital cellular processes and increases its mutations [8]. One of the main factors responsible for the harmful effect of ROS on the skin is long-term exposure of the skin to ultraviolet (UV) radiation [34]. UV is assumed to be one of the most harmful environmental factors causing DNA damage [35,36]. Other factors include environmental pollution, smoking, and an incorrect diet. All these factors lead to faster skin aging, inflammatory processes, or even skin cancer [9].

The plants supplied to the body contain antioxidative compounds that control oxidative stress and may prevent many dermatological diseases as well as faster skin aging [33,37]. EA may be such a plant, with proven antioxidant activity in many studies [2,7,9,11,38,39,40,41]. The analysis of the antioxidant potential of this plant was based most often on the evaluation of this parameter in water or alcohol extracts, using commonly used methods of scavenging free radicals, such as 2,2-diphenyl-1-pikcrylhydrazyl (DPPH) [42], 2,2-azinobis (3-ethyl-benzotiazoline-6-sulfonic acid) (ABTS) [2,7,9,11] as well as ferric reducing antioxidant power (FRAP) [2,7]. However, due to the confirmed high correlation between the antioxidant activity and the polyphenols content in the plants, the assessment of total polyphenol content (TPC), most often determined by the Folin–Ciocalteu (FC) method in EA, was frequently performed [7,12,25,43,44,45].

The high antioxidant activity of EA may contribute to wound healing. Karakaya et al. report that the EA extract applied to wounds in rats and mice resulted in their faster healing, which was related to the high antioxidant activity of extracts from this plant. The measurements of total antioxidant status (TAS) and total oxidant status (TOS) in tissues showed that the oxidative stress after using the EA extracts was relatively low as compared to other varieties of this plant, i.e., *Epilobium hirsutum* and *Epilobium stevenii* [25]. EA extracts are effective against harmful superoxide radicals (O_2_∙^−^) and hydroxyl radicals (OH∙^−^), which are very toxic to cells and tissues. These radicals can initiate the production of ROS. The hydroxyl radical (OH∙^−^) can bind nucleotides in DNA, causing strand breakage, which contributes to carcinogenesis, mutagenesis, and cytotoxicity. Consequently, it can cause oxidative damage to DNA, lipids, and proteins. The high correlation between the scavenging of superoxide (O_2_∙^−^) and hydroxyl (OH∙^−^) radicals by EA extracts and the content of phenolic compounds in this plant was shown [46], and such a relationship undoubtedly has an impact on the protection of the skin cells. For example, the skin fibroblasts irradiated with UVB produce hydrogen peroxide (H_2_O_2_) and then hydroxyl radicals (OH∙^−^), which in turn damage their cells [47]. Furthermore, the EA leaf extracts also increased the activity of superoxide dismutase (SOD), which is one of the basic endogenous antioxidant defense mechanisms and is an enzyme that plays a key role in the first stage of anti-radical defense [9,39,48]. It is commonly assumed that the high antioxidant activity of this plant results from its rich chemical composition, mainly the polyphenolic compounds, which act, among others, as strong reactive oxygen scavengers in skin cells [33,49].

**Figure 4:**
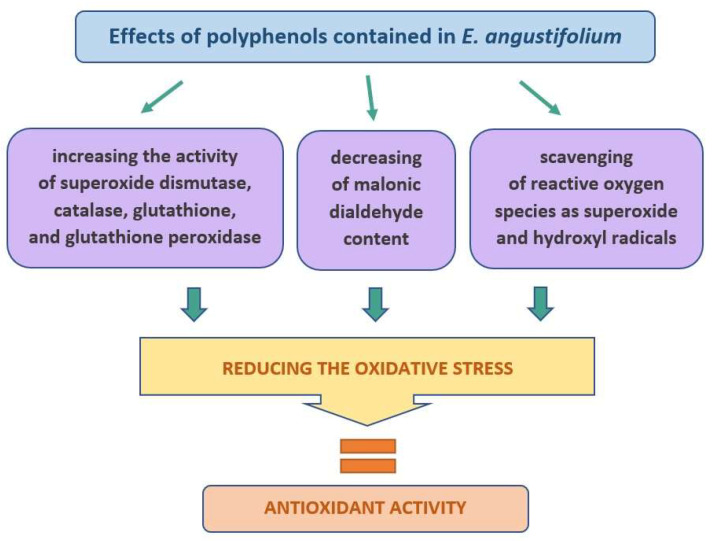
A diagram showing schematically the mechanism of antioxidant activity of the ingredients contained in *E. angustifolium*.

The evaluation in vitro of preparations (hydrogel and emulsion) containing either the dry extract of EA or ethanol extract (70% *v*/*v*) applied to the skin showed that phenolic acids not only permeate through the human skin but also accumulate in it [8,12]. It has also been shown that the skin collected after 24-h penetration also shows an antioxidant effect, as assessed by DPPH and ABTS methods [12]. The accumulation of phenolic acids and their antioxidant effect on the skin can be very important in the case of cosmetic preparations, where a long-lasting effect of the preparation in the skin is desired, and the penetration of the substance into the deeper layers is not so important, or even not advisable [50]. One of the main active substances responsible for the high antioxidant activity of EA is oenothein B (OeB), belonging to the tannins [17,18,51,52]. The OeB increases the level of intracellular reactive oxygen species (ROS) [53]. Li et al. assessed the effect of OeB on the metabolic pathways related to the antioxidant capacity of *Caenorhabditis elegans.* It has been observed that OeB protects RAW 264.7 macrophages from oxidative damage by increasing superoxide dismutase (SOD) activity, catalase (CAT) activity, and glutathione (GSH) content, while decreasing the content of malonic dialdehyde (MDA). In addition, an increase in enzyme glutathione peroxidase (GPx) activity in *C. elegans* was observed [54]. On the other hand, Karakaya et al. suggest that the main component of EA responsible for its antioxidant effect is hyperoside, which has shown a direct effect on faster wound healing [25] (Figure 4).

### 4.2. Anti-Inflammatory Activity

Inflammation is the damage to the normal skin barrier and is often a response to pathogens or trauma and can lead to tissue damage [3]. Inflammatory skin diseases are common dermatological conditions and have a significant impact on the quality of life. The skin inflammatory response is frequently modulated through MAPK, NF-κB, and PI3K/Akt signaling pathways, which contribute to the activity of immune cells and the production of pro-inflammatory cytokines such as interleukin (IL)-1β, IL-6, IL-8, and tumor necrosis factor α (TNF-α) [55,56,57,58,59]. The inflammation is also associated with the generation of excessive oxidative stress caused by external factors such as ultraviolet (UV) irradiation and various types of xenobiotics that disrupt the structure of cellular proteins and the internal redox state [58,60]. The anti-inflammatory action is a necessity for rapid and proper wound healing. The wound healing process consists of the following four basic phases: (1) hemostasis, (2) inflammation, (3) proliferation, and (4) maturation [25]. Since medicinal plants are characterized by a wealth of secondary metabolites, they are more and more often proposed as anti-inflammatory agents. It is believed that in many cases, they could replace nonsteroidal anti-inflammatory drugs (NSAIDs) in slowing down both the causes and the inflammatory effects of the skin [8,61,62].

EA has been used for the healing of wounds in folk medicine [18,25,63]. For a long time, the dried EA herb has been very popular for making anti-inflammatory teas [17]. Many attempts have been made to analyze the extracts of this plant in terms of their anti-inflammatory effects on the skin. For example, the water-ethanol extract of EA inhibited the enzyme lipoxygenase (LOX) by 68%, which may be of great importance in the context of dermatitis [8]. The LOX is an oxidative enzyme with an active non-heme iron atom in its active site and is involved in regulating the inflammatory response. In addition, the LOX catalyzes the oxidation of polyunsaturated fatty acids such as linoleic and arachidonic acids, which results in the initiation of further biological reactions and activation of various cell signaling mechanisms [64]. The potential anti-inflammatory properties of EA were also assessed by the ability to inhibit the denaturation of bovine serum albumin (BSA). The study showed that the concentration of plant extract of 1000 µg/mL can inhibit BSA denaturation from 61% to 67% [8]. The protein denaturation significantly affects its spatial structure and the loss of its biological properties. Therefore, the ability of the plant substances to prevent protein denaturation can also help to prevent inflammation [65]. In other studies, the EA completely lowered 8-iso-prostaglandin F2α (8-iso-PGF2α), which is the isomer of classical prostaglandins. It is produced mainly by oxidative stress-mediated pathways, including reactive oxygen/nitrogen species (ROS/RNS) [21].

**Figure 5:**
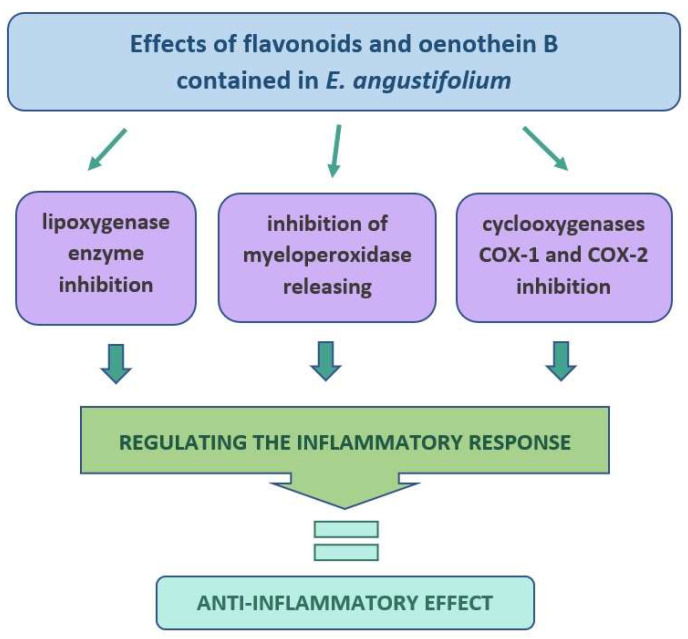
A diagram showing schematically the mechanism of the anti-inflammatory effect of the ingredients contained in *E. angustifolium*.

The certain secondary metabolites found in this plant may be responsible for the anti-inflammatory effect of EA, among others, the OeB, which is a very strong inhibitor of hyaluronidase. The OeB also inhibited very strongly the release of myeloperoxidase (MPO) from stimulated neutrophils [17]. The excessive activity of neutrophils causes a rapid increase in the extracellular formation of reactive oxygen species, such as O_2_∙_−_ and H_2_O_2_, as well as proteolytic enzymes (e.g., elastase) and other inflammatory mediators (e.g., myeloperoxidase, leukotriene B4). The neutrophils are one of the main immune cells in wounds that protect against infection by secreting peptides and enzymes [17,66,67]. The OeB is also a specific inhibitor of the cyclooxygenases COX-1 and COX-2 [17]. The other flavonoids identified in EA are likely to have a little anti-inflammatory effect as compared to OeB [17]. Some authors also reported the anti-inflammatory activity of other compounds found in EA. The myricetin 3-O-β-D-glucuronide, isolated from EA leaves, showed an anti-inflammatory effect on carrageenan-induced swelling of the rat hind paw [64]. The promising active substances also seem to be myricetin-3-O-rhamnoside and myricetin-3-O-glucuronic acid [68], as well as quercetin-3-O-glucuronide and myricetin-3-O-rhamnoside [69]. Karakaya et al. showed that hyperoside could be the compound responsible for wound-healing due to its significant anti-hyaluronidase, anti-collagenase, and antioxidant activities, which are important mechanisms for the wound healing process [25] (Figure 5).

### 4.3. Anti-Aging Effect

Skin aging is a complex biological process influenced by both endogenous and exogenous factors. Among the endogenous factors, the most frequently mentioned are genetic conditions, hormonal processes, and cellular metabolism, while the exogenous factors include exposure to light, pollution, and ionizing radiation, but also to toxins and chemicals. All these factors lead to structural and physiological changes in each layer of the skin [70]. More and more popular is the use in anti-aging preparations of natural ingredients, including plant extracts. The plants, due to their richness in valuable substances, can act on the skin in various ways; among others, they can affect fibroblasts responsible for the production of collagen and elastin. In this case, some of the plant substances can inhibit the enzymes elastase and collagenase, which play a key role in skin aging. An increased activity of proteolytic enzymes, such as metalloproteinases (MMPs), especially matrix metalloproteinase-1 (MMP-1, collagenase-1), matrix metalloproteinase-3 (MMP-3, stromelysin-1), matrix metalloproteinase-8 (MMP-8, collagenase-2), and matrix metalloproteinase-13 (MMP-13, collagenase-3) occurs during skin aging [18,71]. In naturally aging skin in the elderly, the level and activity of MMPs are much higher than in the skin of younger people [72]. However, a very important factor causing skin aging is also excessive ultraviolet (UV) radiation, which leads to changes in the composition of the skin, primarily to the degradation and reduction of collagen, which in turn damages the structure and function of the skin. An additional factor here is the oxidative stress from UV radiation in sunlight, which contributes to skin sunburn and photoaging [18,46].

**Figure 6:**
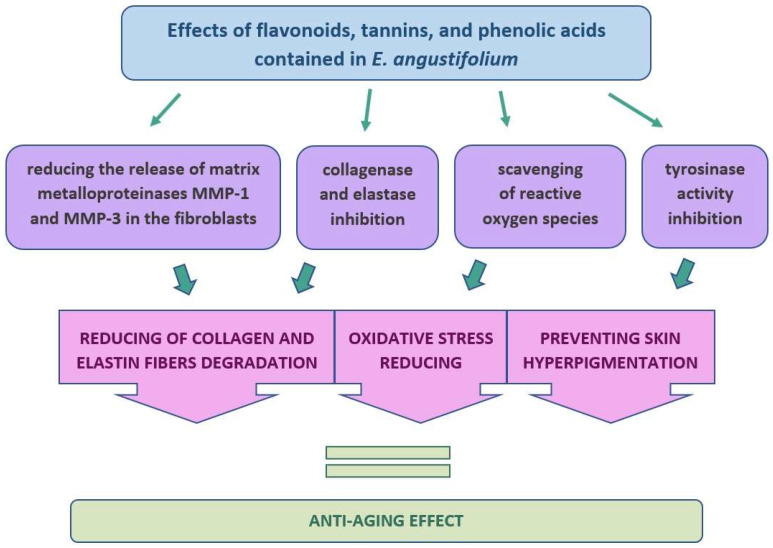
A diagram showing schematically the mechanism of the anti-aging effect of the ingredients contained in *E. angustifolium*.

Due to its valuable composition and high biological activity, the EA may be a potential component of anti-aging preparations [8,9]. The aqueous extract of EA inhibited elastases by 82% [46], while the water-ethanol extract of EA by 48% [8]. The main inhibitory compounds of this enzyme are probably polyphenols, as evidenced by the high correlation between phenolic compounds and anti-elastase activity [46]. In the studies of Karakay et al., the water-acetone extract of EA also markedly inhibited the elastase. The authors, after isolating individual compounds from the extract, showed that the strongest inhibitory effect on this enzyme was caused by hyperoside (inhibition of 20%), then kaempferol (15%), while inhibition of elastase below 10% was shown by such compounds as kaempferol-3-O-α-L-rhamno pyranoside, quercetin-3-O-α-L-rhamno pyranoside, and quercetin-3-O-α-L-arabino pyranoside. The hyperoside also turned out to be the most effective in inhibiting the collagenase enzyme. In this case, the inhibition of activity was 30%, while for the other active substances analyzed it was as follows: 26% for kaempferol, 14% for kaempferol-3-O-α-L-rhamno pyranoside, and 11% for quercetin-3-O-α-L-arabino pyranoside [25]. The anti-aging effect of cosmetic preparations is also aimed at preventing skin hyperpigmentation. For this purpose, tyrosinase inhibitors are used, which inhibit the overproduction of melanin. However, some studies report that synthetic tyrosinase inhibitors are likely to have chronic cytotoxic and mutagenic effects in humans [46]. Therefore, the use of natural substances with fewer side effects is becoming more and more popular. This potential was also shown by the EA aqueous extract, which inhibited the tyrosinase activity by about 15%. In this study, the inhibition of tyrosinase was also correlated with the content of polyphenolic compounds in this plant [46]. In other studies, the treatment of normal human skin fibroblasts (NHFD) (irradiated earlier with UV light) with the alcohol extract with EA reduced the release of MMP-1 and MMP-3 matrix in NHDF. Moreover, complete inhibition of the expression of the enzyme hyaluronidase-2 (Hyal-2), previously induced by 120 h of irradiation, was observed. The inhibition of the Hyal-2 enzyme expression was higher compared to the control (1,3 butanediol, 80% *w*/*w* in water). In this case, the control only partially reduced the amount of the Hyal-2 and showed a very weak antioxidant effect, unlike the EA extract, which was a very strong antioxidant [18]. The inhibition of MMPs activity is influenced by secondary metabolites contained in EA, such as flavonoids, tannins, phenolic acids, terpenoids, and tocopherols [8,24,71]. The specific effect of these compounds is primarily caused by their structure. For example, the presence of carbonyl and hydroxyl groups in flavonoid molecules allows them to form complexes with ions of various metals. As a result, they can also bind to MMPs such as elastase and collagenase and significantly affect various metabolic pathways in the body [73]. It should be noted, however, that the activity of the extract cannot be attributed to the action of a single compound or even one class of compounds, because the stimulating or inhibitory effect of the tested MMPs may be the result of synergism or antagonism between individual plant compounds [74] (Figure 6).

### 4.4. Antimicrobial Activity

The treatment of skin infections by applying plants has been known since antiquity. The secondary metabolites of plants are believed to have a very broad spectrum of antimicrobial activity. Despite the large number of active substances found in the plants, the plant extracts often show a lower antimicrobial potency compared to the pure compounds [75]. However, due to the increasing drug resistance as well as the very common side effects of antibiotics, natural drugs are increasingly popular and can often support antibiotic therapy and, in some cases, even replace it. Moreover, natural drugs are generally safer and less toxic to animal cells than antibiotics [75]. The human skin is an environment for the growth of microorganisms [76]. The main strains of the Gram-positive bacteria found on the skin are *Staphylococcus, Corynebacterium* spp., *Micrococcus*, *Staphylococcus aureus*, and *Streptococcus pyogenes*, which may be dangerous and pathogenic to this tissue, while the Gram-negative bacteria, such as *Pasteurella multocida*, *Pseudomonas aeruginosa*, *Capnocytophaga canimorsus*, *Bartonella* sp., *Klebsiella rhinoscleromatis*, and *Vibrio vulnificus*, are not typical pathogenic of skin microflora; however, with impaired immunity, they can induce various skin infections [76,77]. In recent years, the development of natural methods to combat bacterial skin infections has become increasingly important. It is known that the use of antibiotics contributes to an increased incidence of resistant bacterial strains. In addition, antibiotics can also show many side effects. The bacterial infections are also associated with ROS, which significantly affects the condition of the skin, causing/maintaining inflammation as well as the development of infection [12]. For example, common skin infections, as well as underlying tissue infections, are heavily dependent on oxidative stress [78]. Therefore, plant antioxidants play a key role. The antimicrobial activity of EA has been confirmed in several studies, as shown in Table 1. In most cases, strains such as *Staphylococcus aureus*, *Escherichia coli*, *Pseudomonas aeruginosa*, and the strains belonging to the group *Bacillus* were sensitive to the EA extracts.

**Figure 7:**
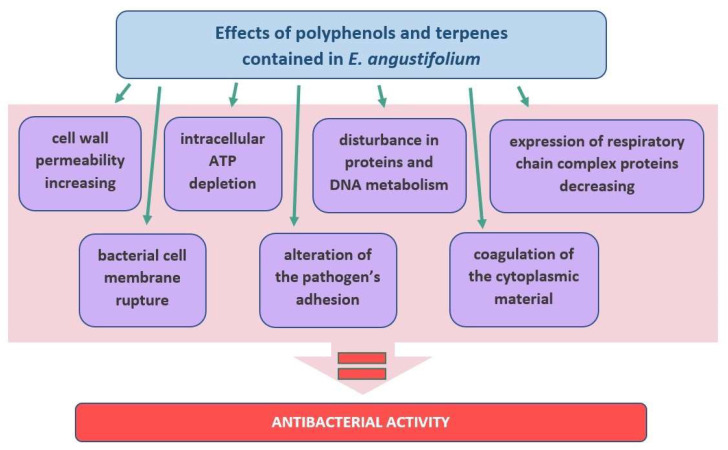
A diagram showing schematically the mechanism of antibacterial activity of the ingredients contained in *E. angustifolium*.

The secondary metabolites of plants play an important role in the fight against bacterial infection of the skin [11,79]. In the case of EA, they are polyphenols [21] and terpenes [23,24]. The most common mechanisms of the antimicrobial action elicited by these compounds are based on the following: (1) cell membrane and cell wall permeability increase [80]; (2) intracellular ATP depletion; (3) inhibition of expression of respiratory chain complex proteins; (4) disturbance in protein and DNA metabolism [81]. For example, carvacrol, the component of EA essential oil [24], which has a phenolic OH group, can cross the bacterial plasma membrane. The carvacrol can bind to molecules such as ATP or monovalent cations such as K^+^ and transport them outside of the bacterial cell [82]. The rupture of the bacterial cell membrane can also be caused by other terpenes found in the essential oil of the EA, such as camphor, citronellol, eugenol, linalool, terpineol, terpinene-4-ol, and thymol [82]. The negative effect of terpenes on the pathogen cell membrane may be based on the modification of the lipid profiles. This increases saturated fatty acids, resulting in an increase in cell membrane stiffness, which, in turn, leads to depolarization, loss of its integrity, reduction of respiratory activity, and, as a consequence, the coagulation of the cytoplasmic material [82]. Another known mechanism of the antimicrobial action of the secondary metabolites is the alteration of the pathogen’s adhesion to the animal cells. The bacterial adhesion to the host surface is essential in the pathogenesis of almost all infectious diseases. It involves the attraction of cells to the surface, followed by absorption and adhesion, followed by molecular reactions between the surface structures of the bacteria and the surfaces of the host’s cell membranes [75,83,84,85] (Figure 7).

### 4.5. Toxicological Study

#### 4.5.1. In Vitro Study

The potential dermatological application of the extract requires evaluating its safety of use against skin cells. Most of the in vitro studies are focused on the proliferative activity based on the MTT assay or cytotoxicity evaluation by using the LDH assay. According to available data, the biological activity of the analyzed plant is strictly dependent on the type of extracts and tested cells [4]. Zagórska-Dziok et al. report that ethanolic extracts of EA in concentrations from 10 to 1000 µg/mL did not show any cytotoxic activity against skin cells but, on the contrary, increased cell proliferation in a dose-dependent manner markedly [9]. Namely, at the concentration of 1000 μg/mL, the fibroblasts (BJ)/keratinocytes (HaCaT) viability ranged from 135% to 145% (depending on the cells and assay used). Moreover, EA extract showed the most promising results from all tested Ayurvedic plant extracts (*Centella asiatica* L., *Clitoria ternatea L.*, and *E. angustifolium*). Authors supposed that this activity may be connected with the presence (only in the EA extracts) of gallic and caffeic acid derivatives, i.e., the compounds with confirmed ability to improve skin cells proliferation and regeneration [86,87,88]. The herbal plant extracts rich in flavonoids, tannins, and phenols facilitate the wound healing process by proliferation and mobilization of fibroblasts and keratinocytes and promote angiogenesis at the site of injury [87]. Ruszová et al. reported a protective effect of 10 μg/mL of EA (in 80% 1.3-butanediol) on the viability of senescent primary normal human dermal fibroblasts (NHDF) induced by serum deprivation [18]. It should be emphasized that, despite the confirmed photoprotective activity, no effect on cell viability was observed when senescence was induced by UVB irradiation. In turn, water extracts from *E. angustifolium*, *E. hirsutum*, and *Epilobium parviflorum* tested on NHDF were not toxic in the concentration range from 3.125 to 50 μg/mL [17].

Contrary to the aforementioned data, the strong antiproliferative activity of EA extracts was observed against human normal and cancer prostate cells, justifying its traditional use in urogenital diseases [88,89]. Depending on the species (*Epilobium rosmarinifolium*, *Epilobium spicatum*, and *Epilobium tetragonum*), the IC_50_ coefficient varies from 80 to 350 µg/mL, 100–780 µg/mL, 40–250 µg/mL, and 200–1200 µg/mL against PZ-HPV-7, LNCaP, normal mammary epithelial cells (HMEC), and astrocytoma cells (1321N1), respectively. According to the authors, the highest antiproliferative activity of *E. rosmarinifolium* was attributed to the highest amount of water-soluble OeB in this extract. On the other side, the antiproliferative activity of the nonpolar fraction (consisting of flavonoids, macrocyclic ellagitannins, and sterols) from all extracts may be responsible for the observed inhibition of the PZ-HPV-7 cell cycle progression. Moreover, Kiss et al. revealed that EA and OeB showed weak but statistically significant inhibition of the proliferation of androgen-independent prostate cancer cells (PC-3). Moreover, the long-term treatment (4 days) of PC-3 cells with EA and OeB resulted in up-regulation of neutral endopeptidase (NEP)-metallopeptidase, whose levels decreased in an early premalignant phase of prostate cancer and stromal benign prostate hyperplasia (BPH) [90]. The continuation of the study on aqueous and methanolic extracts of EA revealed that human neuroblastoma cells (SK-N-SK) with high NEP expression were much more sensitive (IC_50_ between 25 and 50 µg/mL) than PC-3 cells with low NEP expression (IC_50_ > 100 µg/mL) [91]. In general, commercially available ethanolic extracts with low OeB concentrations (0.03–1.3%) may be responsible for the lower antiproliferative activity of EA (IC_50_ reaching 100–780 µg/mL). Furthermore, experiments performed with an aqueous extract of *E. hirsutum* and *E. parviflorum* containing ~23% of OeB demonstrated higher activity, with an IC_50_ against LNCaP yielding 32.2 ± 5.6 µg/mL and 37.3 ± 1.9 µg/mL, respectively. In contrast, aqueous extract from EA (with 15.7 ± 0.1% of OeB) showed lower activity (IC_50_ = 44.6 ± 7.3 µg/mL). The continuation of the study revealed that all aqueous extracts, in a concentration-dependent manner, induce apoptosis in LNCaP cells via disruption of the mitochondrial membrane potential and activation of caspase-3. However, according to the authors, the presence of necrotic cells suggests that additional cytotoxic mechanisms associated with the extensive production of ROS cannot be excluded [89,92]. Similarly, Pei et al. showed that OeB effectively (IC_50_ = 25.49 ± 2.11 μM) inhibited the proliferation of human non-small lung cancer cells (A549) by inducing apoptosis and arresting cells in the G1 phase. Furthermore, OeB significantly (as compared to untreated control) increased the level of intracellular reactive oxygen species (ROS) and intracellular apoptotic markers (cleavage caspase-3, PARP, cytochrome c level in the cytosol, Bax, p53). Finally, the authors suggest that OeB may inhibit cancer cell proliferation via the ROS-mediated PI3K/Akt/NF-κB signaling pathway [53]. The above results seem to be particularly important from the perspective of future application of EA or OeB in skin cancer treatment because PI3K/Akt and also mitogen-activated protein kinase (MAPK) pathways seem to be an attractive target in anti-melanoma therapy [93]. Currently, no data are available on the activity of the crude EA extracts on melanoma cells; nevertheless, they are rich in secondary metabolites (quercetin, kaempferol) with proven activity against skin cancer cells [94,95]. For instance, quercetin (10–40 μM) in a dose-dependent manner inhibited PI3K/Akt and MAPK signaling pathway in UVB-irradiated B16F10 melanoma cells [96], whereas kaempferol targeting mTOR/PI3K/AKT/ pathway leading to cell cycle arrest in G2/M phase, apoptosis and inhibition of cell migration [97].

The results obtained by Hatefi Kia et al. in the experiments on normal (HEK293) and cancer (MCF7) cells proved high antiproliferative selective activity against tumor cells. Interestingly, methanolic extracts were more active than aqueous; moreover, the methanolic extracts derived from the plant roots were more active (IC_50_ = 73 μg/mL) than those from the flowers and aerial parts (IC_50_ equal 110 μg/mL and 160 μg/mL, respectively) [98]. Similarly, different activity between normal and cancer cells were observed in the human colon adenocarcinoma cell line (HT-29) and human colon epithelial cell line (CCD 841 CoTr) after their 96-h treatment with in vitro digested fireweed extract. Even at a low concentration (25 µg/mL), EA inhibited the proliferation of the HT-29 cells to about 50%, and simultaneously dose-dependent stimulation of cell proliferation was noticed in CCD 841 CoTr to 128% at 125 µg/mL [99]. The inhibition of growth and proliferation of human hepatocellular carcinoma cells (HepG2) [1] and some breast cancer cell lines (MCF7, MDA-MB-468, and MDA-MB-231) exposed to aqueous extract of EA was also reported [42]. Moreover, the fraction containing an enhanced amount (91%) of OeB was the most active.

#### 4.5.2. In Vivo and Clinical Study

The information about in vivo and clinical use of EA is very limited. The first in vivo study on the safety of the EA extract took place in the 70s of the XX century and was related to the complex of polyphenols isolated from the blossoms of fireweed [100]. The authors demonstrated that the LD50 value depends on the route of administration and ranges from 10 to 20 mg/kg of mouse body weight after intravenous or intraperitoneal administration, respectively. In turn, Tita et al. revealed that after subcutaneous administration the LD50, was much higher (1.4 ± 0.1 g/kg of mouse body weight) [101]. Roman et al. reported that no signs of toxicity were observed in the brain, hypothalamic-hypophyso-adrenal axis, liver, kidneys, spleen, and thymus of rats treated (intragastrically) with repeated-doses of hydro-alcoholic extracts of *E. hirsutum*, *E. angustifolium*, and *E. parviflorum* [102].

The in vivo studies on the use of EA extracts on the skin are even rarer. No skin irritancy was observed at the highest dose of EA extract (5%), which was one-point applicated under the adhesive tape and left in place for 4 h (patch test). Moreover, EA extract showed a significant photoprotective effect after repeated application of 3 % EA on the skin of healthy human volunteers, before single UV exposure and three-point post-UV exposure treatment [18]. Among the analyzed methanol extracts of *E. angustifolium*, *E. stevenii*, and *E. hirsutum*, the ethyl-acetate sub-extract (EtOAc) of the EA displayed the highest activity during in vivo excision and incision wound mice/rats models. Moreover, it was found that this process is connected with anti-hyaluronidase, anti-collagenase, and antioxidant activities [25].

#### 4.5.3. Side Effects and Interactions of EA

There are not many reports in the literature on side effects or interactions of the extracts containing EA. The richness of active ingredients in the plants can cause adverse effects on the skin, such as allergic and contact dermatitis [103]. It is believed that natural raw materials are safer when applied to the skin when compared to synthetic substances. EA has a weaker anti-inflammatory effect than acetylsalicylic acid. However, this plant may be the basis for perceiving it as a potential raw material with anti-inflammatory properties [8]. Because non-steroidal anti-inflammatory drugs can have several side effects, EA may prove to be a weaker but safer alternative. In addition, there is a great chance for synergism between the active ingredients contained in plants and for obtaining a much better effect when using whole-plant extracts because isolation of individual components may lead to a decrease in this effect. Such synergism has been confirmed in various studies, including anti-inflammatory studies (NSAIDs) [61,104].

## 5. Conclusions

*E. angustifolium* is a plant that has been used for a long time for various skin diseases. The current literature review confirms that EA can be a component of cosmetic or dermatological preparations. The conducted research most often concerns the evaluation of alcoholic or water extracts from this plant, which confirms the antioxidant, anti-inflammatory, antibacterial, and anti-aging effects, which in the context of skin application is very important. Unfortunately, the number of studies on the possibility of using this plant in ready-made dermatological or cosmetic preparations is small, while some of the analyses, e.g., regarding the skin cancer of the skin effect, seem to be insufficient. In the literature, there has been confirmed the therapeutic properties of not only E. angustifolium extracts but also the pure compounds isolated from it. OeB increases the level of ROS as well as protects cells against oxidative damage by increasing the activity of SOD, CAT, and GSH content. OeB is also a compound that affects wound healing and alleviates inflammation by strongly inhibiting hyaluronidase enzymes and inhibiting COX-1 and COX-2 cyclooxygenases. Other compounds of great importance in the context of skin use are the entire pool of polyphenols, mainly flavonoids and phenolic acids. Polyphenols are primarily responsible for reducing oxidative stress, as evidenced by the strong correlation between their content and antioxidant activity. Some flavonoids, including myricetin 3-O-β-D-glucuronide or hyperoside, are promising compounds that may have an impact on faster wound healing and anti-aging effect. They show a strong anti-inflammatory, anti-hyaluronidase, and anti-collagenase effect. Whereas the phenolic acids, such as the gallic and caffeic acid derivatives, show the ability to improve skin cell proliferation. The terpenes in EA, on the other hand, play an important role in fighting bacterial skin infections. These compounds cause, among other things, cell membrane permeability increase as well as the modification of the lipid profiles and the alteration of the pathogen’s adhesion to the animal cells. Due to the richness of secondary metabolites, EA can be seen as a nice candidate for its inclusion in dermocosmetics with multifunctional effects. What is more, EA extracts, as well as the isolated pure compounds, do not show great toxicity to skin cells. Literature data reports indicate the low toxicity of EA extracts towards fibroblasts. On the other hand, in the literature, there is too little in vivo testing on the application of this plant to the skin. Therefore, a continuation is warranted study on the use of EA as an ingredient of dermocosmetics and, in particular, its safety on the skin.

## Figures and Tables

**Table 1:** The biological activity of *E. angustifolium*.

Plant Material/Extract	Experimental Assay	Effect	Reference
EA herb/70% ethanol extract	DPPH *, ABTS *, FC * assay	Antioxidant activity. DPPH method to 3.68 mmol trolox/dm^3^ extract and 12.98 mmol trolox/dm^3^ extract for ABTS, while the total polyphenol content determined by the Folin–Ciocalteu method was 1.94 mmol gallic acid/L extract.	[12]
EA herb/aqueous, 40%, 70% 96% ethanolic; 40%, 70% 99.8% methanolic, 40%, 70% 99.7% isopropanolic extracts	DPPH assay	Antioxidant activity. The antioxidant activity ranged from 13.42% RSA * aqueous extracts in the fruit ripening stage to 96.77% RSA for samples prepared in 70% ethanol (also fruit ripening stage). The FC ranged from 0.38 gallic acid/g raw material for water extracts, extracted in 30 min (intensive growing stage) to 22.99 gallic acid/g raw material for extracts prepared in 70% ethanol (fruit ripening stage).	[2]
EA herb/70% ethanolic extract	DPPH, ABTS assay	Antioxidant activity. The antioxidant properties were 76% RSA and 88% RSA for DPPH and ABTS methods respectively.	[8]
EA leaves/96% ethanolic extract	DPPH, ABTS, FRAP * methods	Antioxidant activity. EA extract showed a DPPH free radical scavenging value of 11.3%, while the ABTS radical scavenging activity was 19.4%.	[11]
EA stems, leaves, blooms/ 75% methanolic extracts	Radical scavenging activities based on the flavonoid content.	Antioxidant activity. Total radical scavenging activity of identified flavonoids was in ranged from 2.72 ± 0.07 to 8.71 ± 0.29 mg/g of raw material during the massive blooming phase and during the intense growth phase, respectively.	[29]
EA herb/70% ethanolic extract	Inhibition of lipoxygenase activity and protein denaturation.	Anti-inflammatory effect. The effect of the extract on the activity of lipoxygenase and BSA denaturation was dose-dependent. The most substantial inhibition was obtained for the extract at a concentration of 1000 µg/mL, where it reached 68.2% inhibition activity of lipoxygenase and 67.7% for inhibited BSA denaturation.	[8]
EA herb/80% ethanolic extracts	Inhibition of lipoxygenase activity and protein denaturation.	Anti-inflammatory effect. The most effective of the concentrations tested for extract were 500 µg/mL. The plant extract inhibited the activity of lipoxygenase to 70.5%, while at a concentration of 1000 µg/mL was able to inhibit denaturation by 61.5%.	[9]
EA aerial parts/hiperozyd (isolated from concentrated methanolic extract)	DPPH assay, FC, collagenase, and hyaluronidase activity. The wound-healing with linear incision and circular excision wound models were created in rats and mice (in vitro study).	Anti-inflammatory, antioxidant, and wound healing activity. IC_50_ values for isolated compounds were in the range between 89.27 and 30.91 µg/mL, while for methanolic extract 49.67 µg/mL. The hyperoside had significant collagenase and hyaluronidase enzyme inhibitory activities with values between 30.07 and 39.66%, respectively. Significant reduction of the wound surface within 15 days of treatment.	[25]
EA herb/70% ethanolic extract	Inhibition of anti-elastase and anti-collagenase activity.	Anti-aging effect. Using the extract at the concentration of 1000 µg/mL, in which inhibition of the elastase activity by 49.1% and collagenase by 59.8% was achieved.	[8]
EA herb/aqueous extracts	MIC * method. Bactrerial strains: *E. coli*, *P. aeruginosa*, *S. aureus*, *Bacillus cereus.*	Antibacterial effect. All bacterial strains were sensitive to the extract. MIC ranged between 78.74 and 198.42 µg/mL and was highest compared to ciprofloxacin.	[21]
EA herb/aqueous extracts	Inhibition zone diameter methods. *Bacterial streins: S. aureus, B. cereus, E. coli, Candidia* spp.	Antibacterial effect. The bactericidal effect was from ≤ 10 ± 1.1 mm for *E. coli* and *Candida* spp. to 25 ± 2.2 mm for *B. cereus*.	[30]
EA leaves/96% ethanolic extracts	Inhibition zone diameter and MIC method. Bacterial strains: *S. aureus*, *E. coli*, *Salmonella*, *Typhimurium*	The extract in concentration 312 μg/mL created the highest inhibition zone diameter on E. coli (8.0 ± 0.10 mm), followed by *S. aureus* (7.0 ± 0.20 mm) and *S. typhimurium* (6.0 ± 0.22 mm), respectively. The inhibition effect of The inhibition effect on bacterial cultures was low compared to the ciprofloxacin.	[11]
EA herb/70% ethanolic extract	Inhibition zone diameter. Bacterial strains: *Serratia lutea*, *S. marcescens*, *Enterococcus faecalis*, *Enterococcus faecium*, *S. pneumoniae*, *P. aeruginosa*, *P. fluorescens*, *B. subtilis*, *Bacillus pseudomycoides*, *Bacillus thuringiensis.*	All the strains were sensitive to the extract used. The strains of bacteria of the genus *Serratia* turned out to be more sensitive than strains of *Bacillus*. The zone of inhibition ranged from 4 mm for *P. aeruginosa* to 16 mm for *S. lutes*.	[12]

* DPPH—2,2-diphenyl-1-pikcrylhydrazyl; ABTS—2,2-azinobis (3-ethyl-benzotiazoline-6-sulfonic acid), FC—total polyphenol content, FRAP—ferric reducing antioxidant power, MIC—minimal inhibitory concentrations, RSA—radical scavenging activity.

## Data Availability

Not applicable.
